# 179. From Epidemiology of Community-onset Bloodstream Infections to the Development of Empirical Antimicrobial Treatment-decision Algorithm in a Region with High Burden of Antimicrobial Resistance

**DOI:** 10.1093/ofid/ofad500.252

**Published:** 2023-11-27

**Authors:** Darunee Chotiprasitsakul, Akeatit Trirattanapikul, Warunyu Namsiripongpun, Narong Chaihongsa, Pitak Santanirand

**Affiliations:** Faculty of Medicine Ramathibodi hospital, Mahidol University, Bangkok, Krung Thep, Thailand; Faculty of Medicine Ramathibodi hospital, Mahidol University, Bangkok, Krung Thep, Thailand; Faculty of Medicine Ramathibodi hospital, Mahidol University, Bangkok, Krung Thep, Thailand; Faculty of Medicine Ramathibodi hospital, Mahidol University, Bangkok, Krung Thep, Thailand; Ramathibodi Hospital, Mahidol University, Bangkok, Krung Thep, Thailand

## Abstract

**Background:**

More antimicrobial-resistant (AMR) infections have emerged in community settings. Our objectives were to study the epidemiology of community-onset bloodstream infections (BSIs), identify risk factors for AMR-BSI and factors associated with mortality, and develop the empirical antimicrobial treatment-decision algorithm.

**Methods:**

A retrospective cohort study was conducted at a tertiary-care hospital. All positive blood cultures from adult patients at the emergency room and outpatient clinics were identified from 1 Aug 2021-15 Apr 2022. AMR was defined as the resistance of organisms to an antimicrobial to which they were at first sensitive. Risk factors associated with AMR-BSI and factors associated with 30-day mortality were determined. The independent risk factors for AMR-BSI were placed into steps to create an empirical treatment-decision algorithm. C-statistics were calculated. The internal validation cohort was evaluated.

**Results:**

A total of 1,151 positive blood cultures were identified. There were 450 initial episodes of bacterial BSI, and 114 BSIs (25%) were AMR-BSI. Nonsusceptibility to ceftriaxone was detected in 40.9% of 195 *E. coli* isolates and 16.4% among 67 *K. pneumoniae* isolates. A treatment-decision algorithm was developed based on the independent risk factors for AMR-BSI: the presence of multidrug-resistant organisms (MDROs) within 90 days (aOR 3.63; 95% CI 1.95-6.75; *P*< 0.001), prior antimicrobial exposure within 90 days (aOR 1.94; 95% CI 1.08-3.50; *P*=0.03), and urinary source (aOR 1.79; 95% CI 1.06-3.01; *P*=0.03). The positive and negative predictive values were 53.3% (95% CI 45.4-61.1%) and 83.2% (95% CI 80.4-85.6%), respectively. The C-statistic was 0.73. Factors significantly associated with 30-day all-cause mortality were Pitt bacteremia score (aHR 1.39; 95% CI 1.20–1.62; *P*< 0.001), solid malignancy (aHR 2.61; 95% CI 1.30–5.24; *P*=0.01), and urinary source (aHR 0.30; 95% CI 0.11–0.79; *P*=0.02).

Univariable analysis and multivariable analysis of risk factors for antimicrobial resistance

**Figure:**
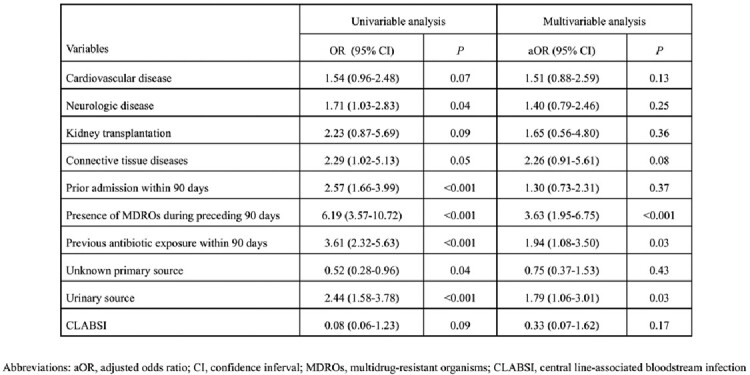

Proposed empirical antimicrobial treatment algorithm for patients with suspected community-onset bloodstream infections

**Figure:**
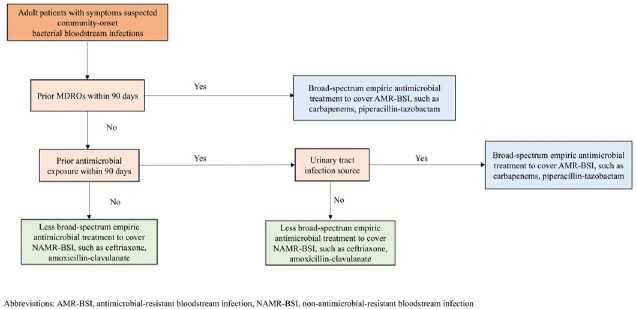

Sensitivity, specificity, positive predictive value (PPV), negative predictive value (NPV), and C-statistics of the algorithm in predicting antimicrobial resistant infection

**Figure:**
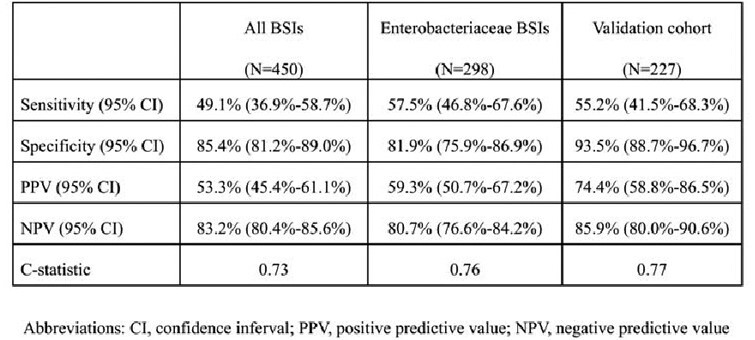

**Conclusion:**

One-fourth of community-onset BSI were antimicrobial-resistant, and almost one-third of Enterobacteriaceae were nonsusceptible to ceftriaxone. Treatment-decision algorithm based on the presence of MDROs within 90 days, prior antimicrobial use within 90 days, and the urinary source may reduce overly broad antimicrobial treatment.

**Disclosures:**

**All Authors**: No reported disclosures

